# The Secretome of Filarial Nematodes and Its Role in Host-Parasite Interactions and Pathogenicity in Onchocerciasis-Associated Epilepsy

**DOI:** 10.3389/fcimb.2021.662766

**Published:** 2021-04-28

**Authors:** An Hotterbeekx, Jolien Perneel, Melissa Krizia Vieri, Robert Colebunders, Samir Kumar-Singh

**Affiliations:** ^1^ Molecular Pathology Group, Laboratory of Cell Biology and Histology, University of Antwerp, Antwerp, Belgium; ^2^ Global Health Institute, University of Antwerp, Antwerp, Belgium

**Keywords:** filarial nematodes, excretory products, onchocerciasis-associated epilepsy, host-pathogen interaction, immune response

## Abstract

Filarial nematodes secrete bioactive molecules which are of interest as potential mediators for manipulating host biology, as they are readily available at the host-parasite interface. The adult parasites can survive for years in the mammalian host, due to their successful modulation of the host immune system and most of these immunomodulatory strategies are based on soluble mediators excreted by the parasite. The secretome of filarial nematodes is a key player in both infection and pathology, making them an interesting target for further investigation. This review summarises the current knowledge regarding the components of the excretory-secretory products (ESPs) of filarial parasites and their bioactive functions in the human host. In addition, the pathogenic potential of the identified components, which are mostly proteins, in the pathophysiology of onchocerciasis-associated epilepsy is discussed.

## Introduction

Filarial nematodes are thread-like worms transmitted by blood-feeding arthropod vectors and affect people from low- and middle-income countries. Adult worms reside in subcutaneous tissue (*Onchocerca volvulus*, *Loa loa*), in the lymphatic system (*Wuchereria bancrofti*, *Brugia malayi*) or in the subcutaneous and connective tissue (*Mansonella* spp.) and produce thousands of small thread-shaped larvae called microfilariae (Taylor et al., 2010). Microfilariae migrate in the skin or blood and are taken up by an arthropod vector during a blood meal (Taylor et al., 2010). It has been shown that the adult filarial parasites can survive for years inside the host by modulating the host immune system through the excretion of soluble mediators (Moreno and Geary, 2008; Specht and Hoerauf, 2009). By playing an important role in both infection and pathology, the secretome of filarial parasites is therefore a promising target for further investigation. Moreover, it has been shown that the anti-filarial drug ivermectin inhibits the excretory-secretory ability of microfilariae, resulting in their death (Moreno et al., 2010; Carithers, 2017; Harischandra et al., 2018; Loghry et al., 2020).

The highest disease burden is associated with lymphatic filariasis (elephantiasis) and onchocerciasis (river blindness). In lymphatic filariasis, dying adults of *W. bancrofti* or *B. malayi* block the lymphatic vessels leading to impaired removal of lymphatic fluids in affected region such as legs, arms, or genitalia. The resulting lymphedema causes swelling and limb disability and is associated with social stigma. In onchocerciasis, *O. volvulus* infection triggers skin disease due to migrating and dying microfilariae, blindness when microfilariae reach the eye, and epilepsy due to an unknown mechanism (onchocerciasis-associated epilepsy or OAE) (Colebunders et al., 2018). There is growing evidence that excretory-secretory products secreted by *O. volvulus* excretory system, the gastro-intestinal tract and reproductive system, as well as proteins shed from the surface of the nematode play a role in various manifestations of onchocerciasis.

This review aims to summarise the current knowledge regarding the various components of the excretory-secretory products (ESP) of filarial nematodes, with a focus on *O. volvulus*, and their bioactive functions in the human host. The review also summarises the current state of knowledge on the pathogenic potential of the identified components in the pathophysiology of OAE.

## Methods

A literature search was conducted until January 27^th^ 2021 using the advanced search option in Pubmed. The search query included excretory OR secretory OR secretome OR excretome OR extracellular vesicle OR exosome AND filarial/Onchocerca. In total 245 articles where retrieved, of which 215 remained after duplicate removal. Articles were screened by two investigators (JP and AH) during a first round based on title and abstract. All articles not discussing ESPs, filarial parasites or targeting diagnostics or immune recognition without going into detail about function were excluded during a first selection round and only research papers were included during a second selection round. Hereafter, 107 articles remained for full text review, of which for seven articles the full text could not be obtained and 69 full text articles were eventually considered for this review. All articles were in English.

## Results and Discussion

### Factors of the Parasite Metabolism and Life Cycle

#### Proteases Play a Key Role in Parasite Migration in the Host Tissue, Transition Through Developmental Stages and Nutrition

Microfilariae and adult males migrate through skin, connective tissue or blood whereas adult females reside in subcutaneous tissue, connective tissue or lymphatic vessels (Taylor et al., 2010). To be able to travel through the host tissue, the connective tissue needs to be broken down and the immune system evaded.

#### Tissue Degradation and Parasite Migration

Proteases and proteolytic enzymes are essential for the survival of filarial nematodes within the host. They are capable of degrading host tissue and immune molecules and are produced in a stage-specific manner, depending on their function (Pokharel et al., 2009). By degrading or interacting with host immune proteins, many nematode proteases may act as enhancers of allergen-induced inflammation and as inducers of Th2 responses. Serine- and metalloproteases that are able to degrade extracellular matrix components such as laminin, collagen type IV and fibronectin, but not IgG, can be detected in the excretory products of *O. volvulus* microfilariae and adult males (Haffner et al., 1998). Furthermore, *O. volvulus* and *B. malayi* infective larvae and to a lesser extent adult females excrete collagenase, which is necessary for invasion of the host tissue during the initial stage of infection (Petralanda et al., 1986). Collagenase helps microfilariae escape from the nodules and migrate through the subcutaneous tissue and increase the chance to be ingested by the vector during a blood meal. It has been suggested that digestion of the collagen in the skin of infested persons over time contributes to the typical skin manifestations of *O. volvulus*, the “leopard” and “lizard” skin (Petralanda et al., 1986).

#### Parasite Development and Nutrition

Collagenase also has an important role in regulating moulting of the larvae, as the cuticle largely consists of collagen (Petralanda et al., 1986). The collagenase produced by infective larvae is immunogenic, leading to protective immunity against new infection in a host with an established infection (Petralanda et al., 1986; Tchakoute et al., 2006). Other proteases such as cathepsin L-like protease and aminopeptidases also have been proposed to play a role in embryogenesis, transition between developmental stages, moulting and cuticle and eggshell remodelling (Guiliano et al., 2004; Pokharel et al., 2009). Cathepsin B-like protease secreted by the bovine parasite *Setaria cervi* was shown to selectively digest bovine haemoglobin and might be involved in nematode nutrition (Pokharel et al., 2009).

Chitinase is produced by the microfilarial stages of *O. volvulus* and *B. malayi* and degrades chitin during larval moulting (Wu et al., 2001; Moreno and Geary, 2008). Although additional immunomodulatory properties have not been attributed to chitinases, an eosinophil chemotactic cytokine has been identified as a chitinase (Moreno and Geary, 2008; Bennuru et al., 2009; Specht and Hoerauf, 2009). Furthermore, the human macrophage chitotriosidase, which is a secreted chitinase, is expressed in a late stage of the differentiation of monocytes to activated macrophages and thus potentially plays a role in the protection against chitin-containing pathogens (Wu et al., 2001).

#### Filarial Parasites Possess Pathways for Sequestration of Host-Derived Fatty Acids

Filarial nematodes are not capable of *de novo* synthesis of complex long-chain fatty acids and are thus reliant on the host for obtaining these. Therefore, filariae possess a variety of pathways for rapid sequestration and incorporation of host-derived fatty acids (Mpagi et al., 2000a). The OvS1/Ov20 secretory protein of *O. volvulus* competes with the host long-chain fatty acid carrier proteins such as serum albumin (Mpagi et al., 2000a). OvS1 in particular shows high affinity for oleic and arachidonic acid whereas Ov20 also sequesters vitamin A (Kennedy et al., 1997; Mpagi et al., 2000a), which might contribute to onchocerciasis-associated eye disease (Kennedy et al., 1997). OvS1/Ov20 bound to fatty acids and retinol can be reabsorbed by the parasite and contribute to the retinol store accumulation, which is essential for parasite metabolism, development and reproduction (Kennedy et al., 1997). Additionally, *O. volvulus* produces a fatty acid- and retinol- binding protein (Kennedy et al., 1997). Fatty acid- and retinol- binding protein may modulate the immune system, as retinol normally prevents the decline in IgA and Th2 cytokine level in the intestinal mucosa (Basavaraju et al., 2003). Furthermore, nematode-derived eicosanoids such as prostaglandins may modulate the host immune response by enhancing the Th2 response and down regulating the Th1 response through IL-10 (Kennedy et al., 1997; Mpagi et al., 2000a; Mpagi et al., 2000b).

#### Cysteine Protease Inhibitors Contribute to the Evasion of the Host- and Vector Immune Response

Filarial parasites encode homologues of cysteine protease inhibitors or cystatins which were identified in all life stages and genders of a range of filarial nematodes (Lustigman et al., 1992; Klager et al., 1999; Manoury et al., 2001; Guiliano et al., 2004). Cysteine protease inhibitors induce immunosuppression and modulate the natural host-parasite relationship by inhibiting a broad range of lysosomal cysteine proteases and by inhibiting host proteases involved in antigen processing and presentation including the key enzyme asparaginyl endopeptidase (Manoury et al., 2001). Additionally, cysteine protease inhibitors also play a crucial role in parasite moulting and embryogenesis. *O. volvulus* and its bovine relative *Onchocerca ochengi* adult females and microfilariae produce onchocystatin, a cysteine protease inhibitor with a potential role in larval moulting and the evasion of the blackfly vector immune system (Klager et al., 1999). Onchocystatin is mainly found in the extracellular space and is highly immunogenic during infection (Lustigman et al., 1992). Blackflies produce cadhepsin L-like cysteine proteases to eliminate invading parasites and simultaneous injection of *O. volvulus* skin microfilariae with onchocystatin improved the parasite survival in the *Simulium damnosum* blackfly vector (Klager et al., 1999). Furthermore, *B. malayi* was shown to produce *Bm*-CPI-2, a cystatin homolog which inhibits multiple cysteine proteases found in human B lymphocytes (Manoury et al., 2001). These proteases are necessary to remove the invariant chain chaperone to generate antigenic peptides presented by MHC class II molecules of the host immune system.

### Strategies to Escape the Host Immune System

**Figure 1:**
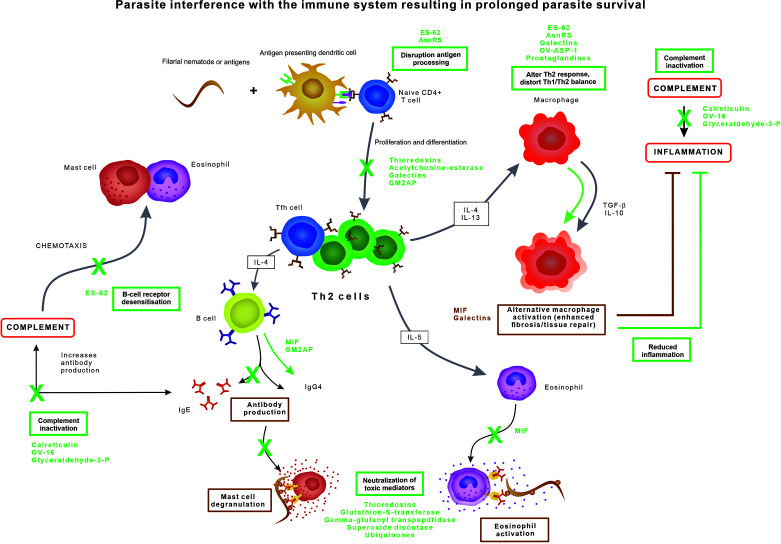
Parasite evasion strategies interfering with the normal immune response, resulting in prolonged parasite survival.

The immune response against nematodes, orchestrated through multiple pathways, results in cumulative damage to the parasite (**Figure 1**) (Maizels et al., 2012). The innate immune system is the first responder to the foreign antigens and the inferred tissue damage when parasites are invading the host tissue. Filarial nematodes have developed three principal strategies to escape the immune system (**Figure 1**): 1) by exploiting and promoting the anti-inflammatory pathways, 2) by neutralizing toxic substances produced by immune cells, or 3) by modulating the effector cells of the immune system.

#### Promotion of the Anti-Inflammatory Pathways

Generally, the immunomodulatory ability of filarial parasites results in an impaired lymphocyte proliferation and polarisation of both cytokine and antibody production. T cells have an essential role in driving the protective immunity against parasites by facilitating and enhancing anti-parasite responses. Helminth infections are dominated by a CD4+ helper T cells type 2 (Th2)-dominant profile as Th2 cells are activated by parasite antigens, leading to the promotion of B cell proliferation and induction of a class switch of B cell antibody production to IgE and IgG4. IgE recognizing parasite surface antigens coats the parasite and induces degranulation of eosinophils and mast cells, leading to the release of chemokines and other toxic compounds to amplify the immune response and kill the parasite (Maizels et al., 2012). Eosinophils and mast cells further drive the Th2-type response by the secretion of IL-4, IL-5 and IL-13 cytokines. However, the inflammatory response of the host does not only cause damage to the parasite but also to the surrounding host tissue. This damage is limited by IL-4 and IL-13 secreted by Th2 cells causes alternative activation of macrophages into the M2 type, which in turn produce the anti-inflammatory and immunomodulatory cytokines TGF-β and IL-10 and promote wound repair and fibrosis (Maizels et al., 2012). There is a suppression of Th1 responses resulting in a reduction of TNF-alpha. The antibody response is dominated by high levels of IgG4 antibodies which are unable to activate complement or bind to phagocytic cells and thus have limited value in eliminating pathogens as they block potential protective activities from other antibody isotypes (Al-Riyami and Harnett, 2012; Pineda et al., 2014).

ES-62** **is probably the most extensive studied excretory protein of parasitic nematodes, with homologues in almost all filarial parasites, including *O. volvulus*. ES-62 is a phosphorylcholine-containing leucyl aminopeptidase capable of interfering with the host immune response in a variety of ways, affecting a variety of immune cell types and mechanisms. In addition to inhibiting complement activation, ES-62 has been shown to modulate key signal transduction pathways involved in immune cell activation and polarisation, as reviewed extensively before (Bennuru et al., 2009; Al-Riyami and Harnett, 2012; Pineda et al., 2014). Briefly, ES-62 desensitises B- and T-cell receptor signalling, the mast cell response and the antigen-presenting cell response. The anti-inflammatory actions of ES-62 are due to its unusual post-translational modification in which the phosphorylcholine unit attached to a terminal N-acetylglucosamine residue.

#### Filarial Parasites Possess Different Mechanisms Interfering With the Complement Activation Pathway

Calreticulin is a Ca^2+^ binding secretory protein produced by all life stages of *O. volvulus* and other filarial parasites such as *Dirofilaria immitis and B. malayi*. The human calreticulin homologue is involved in the regulation of gene expression, cellular Ca^2+^ homeostasis, endothelial nitric oxide production, chaperone activity, interaction with protein disulphide isomerase, and acting as a C1q receptor on the surface of phagocytes (Tsuji et al., 1998; Yadav et al., 2014). Parasite-derived calreticulin inhibits the complement activation pathway by interacting with the first component C1q of the human complement system (Tsuji et al., 1998; Yadav et al., 2014; Abdel-Latif, 2020). C1q is the recognition protein of the classical complement activation pathway which normally exists as part of the C1 complex, and Ca^2+^ is essential for C1q stability and function. The presence of Ca^2+^ promotes strong interaction of filarial calreticulin with C1q, which in turn blocks the release of Ca^2+^ from C1q, thereby blocking proper orientation of C1q and ultimately preventing the activation of the classical complement pathway (Yadav et al., 2014).

In addition, OV-16, a putative serine protease inhibitor and phosphatidylethanolamine-binding protein also used as a diagnostic target for *O. volvulus*, has been identified as an inhibitor of the classical and alternative complement pathways by binding to C9 and C3 (Moreno and Geary, 2008). This results in a reduction of C3 cleavage as well as soluble C5b-9. Prevention of C3 cleavage prevents opsonisation, phagocytosis, complement-mediated cytolysis and inflammation, whereas binding with C9 inhibits formation of the complement membrane attack complex (Abdel-Latif, 2020). The first is probably the most important for survival of filarial nematodes since C3a is neutrophil chemotactic and stimulates the release of inflammatory mediators from various leukocytes (Abdel-Latif, 2020). Furthermore, glyceraldehyde-3-phosphate dehydrogenase has been identified as a C3 binding protein (Abdel-Latif, 2020) and paramyosin was found to bind both C1q and C9 in the roundworm *Trichinella spiralis* and was also identified in *O. volvulus* and *D. immitis* (Sommer et al., 2001; Morchon et al., 2014; North et al., 2019; Abdel-Latif, 2020).

Human serine protease inhibitors or serpins regulate fundamental processes, including coagulation, complement activation and inflammation. However, the potential role of filarial serine protease inhibitors as modulators of host immune responses are not well characterised. SPN-2 from *B. malayi* induces the *in vitro* production of IFN-γ, characteristic of a Th1 response (Moreno and Geary, 2008; Bennuru et al., 2009), but not production of IL-4 or IL-5 in murine T cells. Similar to cystatin homologues, serine protease inhibitors are vital in moulting, embryogenesis and spermatogenesis of the filarial parasite (Moreno and Geary, 2008), however, their immunological consequences have not yet been described.

#### Nematode Macrophage Inhibitory Factor (MIF) Homologues Contribute to Immune Evasion

A homologue of the human pro-inflammatory cytokine macrophage migration inhibitory factor (MIF) are excretory products of *O. volvulus, B. malayi* and *W. bancrofti* (Pastrana et al., 1998; Hewitson et al., 2008; Bennuru et al., 2009; Sharma et al., 2012). In humans, MIF is expressed by T cells, macrophages and eosinophils and influences T cell and NK cell activation. MIF is also involved in immunoglobulin synthesis, endotoxin shock, response to glucocorticoid hormones, regulation of insulin secretion and cellular growth and differentiation. In nematodes, MIF plays a role in immune evasion for adult stages and host invasion for the infective stages (Pastrana et al., 1998; Sharma et al., 2012). The filarial MIF has the capability of modifying the activity of macrophages and monocytes and enhancing the expression of several other pro-inflammatory cytokines such as TNF-α, IL-1, IL-2, IL-8 and IFN-γ (Calandra and Roger, 2003). MIF from *B. malayi* drives macrophages to alternative activation, creating an anti-inflammatory environment leading to an IgG4 dominated Th2 immunity (Sharma et al., 2012). Furthermore, MIF inhibits the NK-cell-mediated cytotoxicity by preventing perforin release and inhibits the random migration of macrophages as well as acting as a chemoattractant (Pastrana et al., 1998; Sharma et al., 2012).

#### Parasite Galectins Bind to Monocytes and T Cells

Galectins are excreted by *O. volvulus* and *B. malayi* and promote *in vivo* parasite survival (Vasta, 2009; Hertz et al., 2020). They may be upregulated in response to oxidative stress. Galectins are capable of altering the response of immune cells by binding to monocytes and T cells, thereby altering the cytokine production and inducing apoptosis. Additionally, galectins can bind to IgE, regulate alternative macrophage activation, inhibit lymphocyte trafficking, and accumulate at the endothelial surfaces promoting endothelial proliferation (Hewitson et al., 2008; Bennuru et al., 2009; McSorley et al., 2013; Hertz et al., 2020). Many mammalian immune cells, including macrophages, dendritic cells, mast cells, B cells and T cells recognise pathogenic galectins, thereby triggering an immune response or regulating immune effector cells. Galectins thus participate in both innate and adaptive immune responses and the effects on the immune response is context dependent. Best understood here, is the ability of galectins to bind glycans on the T cell surface to regulate signaling, activation profile, survival, and cytokine production (Hertz et al., 2020). Galectin-1 and -2 inhibit Th1 cytokine release, promote Th2 cytokine release and drive macrophages to an alternative M2 phenotype (Shi et al., 2018). By mimicking some of the functions of their mammalian counterparts, parasitic galectins may play a role in host-pathogen interactions (Shi et al., 2018).

#### Modulation of Immune Cell Effectors

Thioredoxins are involved in detoxifying Reactive Oxygen Species (ROS) (Ghosh et al., 1998) and are able to inhibit TNF-α induced activation of p38 mitogen-activated protein (MAP) kinase (Sharma et al., 2012). MAP kinase is involved in several key signalling pathways of antigen-presenting cells (APCs) and can thus control binding of transcription factors with immunological activity, such as NF-κβ and AP-1. When activated through the MAPK pathway, AP-1 and NF-κβ promote inflammation, cytokine production, and immune cell proliferation and recruitment. Thus, by uncoupling the MAPK pathway, thioredoxins have the ability to modulate cytokine release, enhance cytotoxicity, block chemokine activity, and protect the parasite against reactive radicals (Kunchithapautham et al., 2003).

Acetylcholinesterase hydrolyses and degrades the acetylcholine neurotransmitter, which is also involved in many immunological processes (Rathaur et al., 1987; Sharma et al., 1998; Sharma and Rathaur, 1999). Despite being well conserved among parasitic nematodes and its presence in the excretory-secretory products of numerous filarial parasites, not much is known about the functionality of this protein at the host-parasite interface (Rathaur et al., 1987; Sharma et al., 1998; Sharma and Rathaur, 1999). Acetylcholinesterase secreted by filarial parasites may interfere with a wide variety of immunological responses by reducing acetylcholine levels. Acetylcholine enhances the release of mediators from mast cells, the cytotoxicity of neutrophils and lymphocytes, the lysosomal enzyme secretion, phagocytosis and antibody-dependent cytotoxicity. Additionally, by elevating intracellular cGMP levels, acetylcholine can also release T cells from their IL-2 dependence to produce IFN-γ and may also interfere with other T cell functions (Rathaur et al., 1987).

Another excretory-secretory protein acting on the innate immune response is asparaginyl-tRNA synthetase (AsnRS), which is highly expressed in all life stages of *B. malayi*. However, data on AsnRS production by *O. volvulus* is as yet lacking. AsnRS has the ability to activate IL-8 receptors and acts as a chemoattractant for primary human lymphocytes, immature dendritic cells, neutrophils and eosinophils by interacting with and activating IL-8 receptors as well as the CXCR1 and CXCR2 chemokine receptors (Ramirez et al., 2006; Kron et al., 2012; Hameed et al., 2019). Additionally, AsnRS blocks CXCL1-induced calcium influx and induces activation of MAP kinases Erk1 and Erk2 involved in the cell migration signal transduction pathway and desensitises the growth regulating oncogene (Gro)–a–induced calcium mobilisation in neutrophils (Ramirez et al., 2006). AsnRS trigger the maturation of IL-10+ regulatory T cells *in vitro* by interacting with immature dendritic cells, by binding to its IL-8 receptor, while AsnRS enhances the expression of IL-4 and IL-10 *in vivo* and induces a shift towards a Th2 cytokine pattern (Ramirez et al., 2006; Kron et al., 2012). Since several other leukocyte subsets including mast cells, NK cells and CD8+ T cells also express CXCR1 and CXCR2 receptors with which AsnRS could also possibly interact (Ramirez et al., 2006).

Cyclophilins are a member of the immunophilin protein family, which is characterised by peptidyl-propyl-cis-trans isomerase activity and are secreted by filarial parasites. They are a component of the cuticle, involved in protein folding, and act as molecular chaperones (Hewitson et al., 2008; Bennuru et al., 2009; Eberle et al., 2015). Whether there is a role for cyclophilins in immunomodulation is unclear, although human cyclophilin A plays a role in leukocyte chemotaxis and immune cell adhesion (Song et al., 2011).A cyclophilin of the protozoan parasite *T. gondii* was found to be directly involved in the host-parasite interplay, stimulating a protective Th1 response by binding to the chemokine receptor CCR5 (Denkers, 2003). Similarly, it might be possible that cyclophilins from filarial parasites may serve a similar function in immune evasion (Hewitson et al., 2008).

Ov-ASP-1, the *O. volvulus* homologue of the activation associated secreted gene family was found to stimulate IFN-γ secretion by PBMC and IL-5 secretion and thus has intrinsic Th1 and, to a lesser extent, Th2-stimulating properties (MacDonald et al., 2004).

Recently, a 28 kDa cysteine-rich protein has been identified as a putative ganglioside GM2 Activator Protein (GM2AP) in *O. volvulus. O. volvulus* GM2AP does not facilitate the degradation of GM2 ganglioside, which is the main function of human GM2AP, but it was shown to inhibit human phospholipase D activity and inhibit fluorogenic β-N-acetylhexosaminidase substrate (4-methylumbelliferyl-2-acetamido-2-deoxy-β-D-glucopyranoside) in a competitive manner with the human orthologue, suggesting the presence of similar functional domains (Njume et al., 2019). In addition, GM2AP has been shown to act as a biological detergent capable of solubilizing, binding and transporting different lipids, but this function has not yet been observed in filarial nematodes (Mahuran, 1998). GM2AP is secreted by *O. volvulus* L3 larvae, adult males and females, and elicits an IgG immune response in infected individuals (Njume et al., 2019). The exact role of nematode GM2AP in immune modulation remains unclear. However, phospholipase D plays an important role in T cell receptor-mediated signalling and cell activation by generating the important signalling lipid phosphatidic acid. Inhibition of phospholipase D has been shown to impair TCR-mediated signalling, GM2AP may thus modulate the T cell response in favour of the parasite (Zhu et al., 2018). Additionally, the GM2AP orthologue form *Trichinella spiralis* was shown to enhance neutrophil chemotaxis, mast cell degranulation and platelet activation factor induced Ca2+ mobilisation (Bruce et al., 2006; Zhu et al., 2018).

Furthermore, filaria-derived eicosanoids may participate in host-parasite interactions and modulate the immune response to the parasite (Kennedy et al., 1997; Mpagi et al., 2000b; Abdel-Latif et al., 2015). Filarial eicosanoids such as prostaglandins enhance the Th2 and down-regulate the Th1 type immune responses and are thought to regulate physiological processes enabling the parasites to disseminate and reproduce in the host (Kennedy et al., 1997; Mpagi et al., 2000b; Abdel-Latif et al., 2015).

#### Antioxidant Enzymes Neutralise Reactive Oxygen and Nitrogen Species

Several filarial parasites produce antioxidant enzymes such as thioredoxin, thioredoxin peroxidases, glutathione S-transferases, glutathione peroxidase and superoxide dismutases (SODs) as protection against the toxic contents released by immune effector cells as a first-line host defence mechanism.

#### Thioredoxin and Thioredoxin-Dependent Peroxidase Protect Against ROS-Induced Stress

Thioredoxins are small redox proteins which can function as reducing equivalents in biological systems and are produced as a defence mechanism against toxic hydroxyl radicals (Kunchithapautham et al., 2003). Thioredoxin is used as electron carrier by anti-oxidant enzymes such as thioredoxin peroxidase, glutathione peroxidase (GPX) and glutathione-S-transferase (GST), which are excreted simultaneously with thioredoxin (Kunchithapautham et al., 2003). These peroxidases are excreted by all life stages of various filarial parasites, including *O. volvulus* and *B. malayi* (Ghosh et al., 1998; Kunchithapautham et al., 2003; Ajonina-Ekoti et al., 2012). GPX is abundant in adults and acts as a lipid hydroperoxidase, protecting the parasite membrane against ROS, and removing host immunomodulatory lipids (Cookson et al., 1992; Singh and Rathaur, 2005; Bennuru et al., 2009).

A sigma-class GST secreted by *O. volvulus* acts as a prostaglandin D2 synthase (Sommer et al., 2001). It has been implicated in the biotransformation of inorganic arsenic, modulation of ryanodine receptor calcium release channels, post-translational processing of IL-1β in monocytes, and drosopterin biosynthesis (Sommer et al., 2001). An omega-class GSTs *of O. volvulus* is involved in the oxidative stress response and plays a role in reversible S-glutathionylation and glutathione-mediated redox regulation of proteins. Thus, glutathione-S-transferases are involved in the metabolism, detoxification and repair mechanisms of filarial parasites (Rao et al., 2000; Sommer et al., 2001; Gupta et al., 2007; Liebau et al., 2008).

Gamma-glutamyl transpeptidase is a multifunctional enzyme involved in glutathione metabolism and is known to modulate the host immune system, it is released by adult females and microfilariae but not by the adult males. Furthermore, it is involved in the catabolism of immunomodulatory and pro-inflammatory cysteinyl-leukotrienes (Hewitson et al., 2008; Moreno and Geary, 2008).

#### Superoxide Dismutase Dismantle Superoxide and Hydrogen Peroxide Released by Host Immune Cells

Superoxide dismutases (SODs) are metalloenzymes that catalyse the conversion of superoxide anion into hydrogen peroxide and molecular oxygen protecting the parasite against endogenous oxidative stress, and are detected in several filarial parasites (Rathaur et al., 2001; Ajonina-Ekoti et al., 2012). SODs may modulate adaptive immunity since H_2_O_2_ can act as a second messenger in lymphocyte activation. Additionally, a probable offensive function of SOD was proposed, in which the H_2_O_2_ would be produced as mobile oxidant directed against the host. Furthermore, there is a potential role for Cu/Zn SODs in nodule formation since the production of H_2_O_2_ stimulates cell proliferation and promotes VEGF signalling in angiogenesis. SODs are detected in the ESPs from all life stages, although the relative abundance of the enzyme in each life stage is not clear (Hewitson et al., 2008; Moreno and Geary, 2008; Bennuru et al., 2009).

Furthermore, two ubiquinones (Q_6_ and Q_8_) have been identified in the ESPs of the filarial parasite *S. digitate* (Mohan et al., 2001). The preferential release of ubiquinone Q_8_ in the ESPs has an antioxidant role by inhibiting lipid peroxidation while the ubiquinone Q_6_ is primarily involved in the electron transport chain (Mohan et al., 2001).

### The Potential Role of Parasite Excreted Factors in Onchocerciasis-Associated Epilepsy

There is currently strong epidemiological evidence for the association between *O. volvulus* infection and the development of epilepsy, referred hereafter as onchocerciasis-associated epilepsy (OAE). The pathological mechanism of this epilepsy is not known, but the level of skin microfilarial density in children determines their risk to develop epilepsy (Chesnais et al., 2018; Chesnais et al., 2020), suggesting a direct effect of the parasite. While detailed reviews on OAE can be found elsewhere (Colebunders et al., 2018), here we aim to review the potential role of *O. volvulus-*derived ESPs in the development of OAE.

#### Disruption of Host Signalling Molecules

Several filarial ESPs are involved in the synthesis and metabolism of eicosanoids, lipids and acetylcholinesterase which degrades the neurotransmitter acetylcholine. By disrupting the normal host metabolism of these compounds, *O. volvulus* may influence neurodevelopment and neural function which may lead to the development of seizures and intellectual disability. Additionally, by influencing hormone levels, *O. volvulus-*derived ESPs have also been proposed to contribute to the bone deformities and lack of sexual maturation seen in Nakalanga syndrome (Colebunders et al., 2018).

Lipid-mediated signalling is involved in many physiological processes, including multiple facets of brain function such as synaptic function and neuroprotection (Oliveira and Di Paolo, 2010; Zhang and Bazan, 2010). Dysregulation of these lipid pathways is implicated in a growing number of neurodegenerative disorders including Alzheimer’s disease (Oliveira and Di Paolo, 2010). Eicosanoids, which include prostaglandins, thromboxanes, leukotrienes and lipoxins, are important lipid mediators playing an role in neural function including sleep induction, long term potentiation, spatial learning and synaptic plasticity, inflammation, and anti-inflammatory and neuroprotective bioactivity (Tassoni et al., 2008). *O. volvulus-*derived eicosanoid homologues and molecules interfering with the human lipid metabolism could thus have a negative influence on the developing brain.

A number of ESPs such as GSTs, ES-62 and OvS1/Ov20 are implicated in eicosanoid metabolism as well as with its precursor, arachidonic acid and filarial parasite may produce their own eicosanoids. Alterations in the GSTs genes have already been implicated in Alzheimer’s and Parkinson’s disease (Schmuck et al., 2005; Rebai et al., 2021).

Human GM2AP functions is a vital cofactor of N-acetylhexosaminidase A to degrade the ganglioside GM2 in the lysosome and solubilises, binds and transports different lipids (Mahuran, 1998). It binds and transports different lipids, playing a crucial role in the central nervous system (Mahuran, 1998; Njume et al., 2019). Without the GM2 ganglioside activator protein, N-acetylhexosaminidase A is unable to break down the GM2 ganglioside which results in toxic build-ups in neuronal cells (Mahuran, 1998). GM2 gangliosidosis is a genetic storage disease where GM2 catabolism is disturbed and GM2 accumulated in lysosomes, such as in Tay-Sachs disease and Sandhof disease (Sandhoff and Harzer, 2013). GM2 gangliosidoses have diverse clinical manifestations but the central nervous system is most affected, with cerebral degeneration, epilepsy, muscle involvement and psychiatric symptoms (Sandhoff and Harzer, 2013). The filarial homologue of GM2AP has been shown to bind the interactors of its human counterpart such as phospholipase D in a competitive manner (Njume et al., 2019). This again raises the possibility that by disrupting lipid signalling and metabolism, the ESPs of filarial parasites may cause significant damage and pathology in the brain. For example, phospholipase D has been implicated in a variety of neurodegenerative diseases such as Parkinson’s- and Alzheimer’s disease and plays important roles, not only in T cell signalling, but also in cellular degeneration and cell survival (Oliveira and Di Paolo, 2010).

Other ESPs such as acetylcholinesterase and aromatic amino acid decarboxylase (AADC) are key enzymes in the synthesis of neurotransmitters which can influence neurotransmitter balance and functioning. Filarial parasites express and secrete a homologue of AADC which is a key enzyme in the metabolism of monoamine neurotransmitters (e.g., dopamine, serotonin, adrenalin) and trace amine neuromodulators (e.g., tyramine, phenethylamine, synephrine) important in neurophysiology. The malfunction of these amines has been associated with neuronal diseases such as schizophrenia and Parkinson’s disease. Other functions of AADC are catalysing the formation of neurotransmitter in non-monoaminergic neuronal-like cells, synthesis of dopamine in kidney, and detoxification of L-amino acids in the liver (Tang and Frank, 2001). Interestingly, *O. volvulus* has been shown to excrete the inactive form of the trace amine tyramine, N-acetyltyramine (Globisch et al., 2013; Globisch et al., 2017).

#### Disruption of Host Immune System

ESPs might be able to cause seizures either by a direct neuromodulator effect, when entering the central nervous system, or by an indirect effect through neuroimmune modulation and neuroinflammation. Since a functional immune system is important for proper neurocognition and neurodevelopment (Johnson et al., 2020), immune alterations caused by the ESPs of *O. volvulus* may disrupt these normal processes resulting in aberrant signalling and ultimately seizure activity in the brain. Especially, the young brain is vulnerable for such processes as it still undergoes remodelling and maturation, which would explain why only children between the ages of 3-18 years develop epileptic seizures and suffer from severe intellectual disability. The role for neuroinflammation in OAE pathology in the brain is supported by the considerable presence of neuroinflammatory cells and reactive tau accumulation as neurofibrillary tangles and threads reported in two post-mortem studies (Pollanen et al., 2018; Hotterbeekx et al., 2019a).

## Concluding Remarks

The secretory products of filarial parasite contain a diverse set of enzymes and proteins implicated in immunomodulation, the metabolism and life cycle of the nematode. We discussed how the secretion of these bioactive molecules could interfere with normal host biology, and described possible mechanisms by which it can cause pathology. While the precise pathogenesis of OAE is unknown, the level *O. volvulus* infection is tightly associated with an increased risk of developing epilepsy (Chesnais et al., 2018; Chesnais et al., 2020). This suggest that there may be a direct effect of the nematode on the central nervous system. However, to date, *O. volvulus* has not been observed in post-mortem brain tissue (Pollanen et al., 2018; Hotterbeekx et al., 2019a), and it is unlikely that microfilariae cross the blood-brain barrier (Winkler et al., 2013; Hotterbeekx et al., 2019b). The presence of human homologues for several parasite ESPs may be implicated in neurological disease and play a crucial role in neurobiology (Liebau et al., 2008; Oliveira and Di Paolo, 2010; Sandhoff and Harzer, 2013). A question that remains to be addressed is why only the ESPs of *O. volvulus* and not of other filarial parasites would produce epileptic activity. Consequently, differences in ESPs produced by *O. volvulus* and other filarial parasites should be investigated to identify unique *Onchocerca* bioactive neurotoxic compounds.

## Author Contributions

AH and JP: literature review and drafting manuscript. AH: generation of figure. All authors: adapting and reviewing the manuscript. All authors contributed to the article and approved the submitted version.

## Funding

This review was funded by University of Antwerp (GOA s30729) and European Research Council (ERC 671055).

## Conflict of Interest

The authors declare that the research was conducted in the absence of any commercial or financial relationships that could be construed as a potential conflict of interest.
